# Seasonal summer stress affects systemic redox homeostasis: a longitudinal marker analysis in healthy adults

**DOI:** 10.3389/fpubh.2026.1778888

**Published:** 2026-05-22

**Authors:** Hong Zhou, Jiachen Dai, Yuemei Zhang, Wenwen Tang, Yingying Ren, Xinming Zhang, George H. Lorimer, Hasan Bayram, Reza A. Ghilad, Junqiang Xu, Fanghua Mei, Jun Wang

**Affiliations:** 1 National “111” Center for Cellular Regulation and Molecular Pharmaceutics, Hubei University of Technology, Wuhan, Hubei, China; 2 International Center for Redox Biology & Precision Medicine of Hubei Province, Hubei University of Technology, Wuhan, Hubei, China; 3 Cooperative Innovation Center of Industrial Fermentation, Hubei University of Technology, Wuhan, Hubei, China; 4 Department of Chemistry, University of Maryland, College Park, MD, United States; 5 Department of Pulmonary Medicine, School of Medicine, Koc University, Istanbul, Türkiye; 6 Department of Chemistry, North Carolina State University, Raleigh, NC, United States; 7 Hubei Center for Disease Control and Prevention, Wuhan, China

**Keywords:** antioxidant, malondialdehyde, natural summer stress, nitric oxide, redox marker

## Abstract

**Introduction:**

As global climate change and urbanization accelerate, summer heat waves are becoming more frequent and intense, posing an increasing threat to human health. However, previous studies have predominantly focused on the impact of summer high temperatures on people with pre-existing diseases, with few reports investigating their effects on healthy individuals. Redox balance is a core foundation of human health and is closely linked to changes in ambient temperature.

**Methods:**

The present study enrolled 330 healthy volunteers from four cities in Hubei Province, China. Fasting serum samples were collected during spring (mild temperatures) and summer (high temperatures), and the levels of six redox markers were measured and analyzed: nitrite (NO_2_^−^), nitrate (NO_3_^−^), oxidized glutathione (GSSG), malondialdehyde (MDA), and total antioxidant capacity.

**Results:**

The results revealed that under *natural summer stress*, systemic redox homeostasis in healthy individuals is significantly perturbed, characterized by reduced nitric oxide bioavailability, elevated lipid peroxidation, and enhanced serum antioxidant activity. Notably, MDA was the most sensitive biomarker in response to natural summer stress, with minimal interference from age or sex, suggesting its potential as a reliable biomarker for oxidative damage induced by natural summer stress in healthy individuals.

**Discussion:**

Furthermore, significant methodological biases may arise if the seasonal variations in serum MDA levels are not taken into account when researching in this area. These findings provide a novel direction for investigating the impacts of high summer temperatures on health and may lay the foundation for guiding public health practices.

## 1 Introduction

Driven by global climate warming, urbanization, and industrialization, the intensification of the urban heat island effect has resulted in a substantial surge in the number of individuals exposed to extreme heat. Epidemiological data indicate that summer high temperatures exposure caused approximately 489,000 deaths annually between 2000 and 2019, with Asia accounting for 45% and Europe for 36% of these fatalities (1–3). Notably, when comparing the periods 2000–2004 and 2017–2021, heat-related mortality among adults aged 65 years and older increased by roughly 85% (2, 3). Against the backdrop of these worrying trends, there is an urgent need to investigate the health impacts of hot weather and develop targeted preventive strategies (4).

Oxidation–reduction reactions are essential for sustaining life. Under physiological conditions, cells endogenously produce reactive oxygen species (ROS) and reactive nitrogen species (RNS) as metabolic byproducts, both of which exert pivotal roles in the maintenance of cellular homeostasis (5). However, an imbalance between ROS/RNS production and the capacity of antioxidative defense mechanisms can induce oxidative stress (6). Excessive ROS, in particular, can damage cellular lipids, proteins, and nucleic acids (7). ROS and RNS are regulated by various factors, including seasonal variations. Their dysregulation leads to the pathogenesis of various diseases (8).

Lipid peroxidation, a process involving the oxidative conversion of polyunsaturated fatty acids to malondialdehyde (MDA), is one of the most extensively studied biologically relevant free radical reactions (9) and is widely recognized as a reliable indicator of oxidative stress (10). Accumulating evidence indicates that *natural summer stress* can impact MDA levels in organisms. For instance, summer worker bee’s exhibit higher MDA levels compared to their winter counterparts (11), while MDA concentrations in perinatal dairy cows are also influenced by summer (12). Furthermore, previous study demonstrated that exposure to 35 °C water induces elevated MDA levels across multiple tissues in spotted seabass, suggesting that MDA may serve as a biomarker for assessing prolonged heat stress in this species (Lateolabrax maculatus). In humans, plasma MDA levels display distinct seasonal patterns. Higher levels are observed in winter/spring among patients with myocardial infarction and normolipidemic individuals, whereas lower levels occur in summer/autumn (13). Additionally, serum MDA levels in patients with schizophrenia exhibit a prominent winter-summer rhythm, with significantly higher concentrations in summer than in winter (14). Yet, few studies have specifically investigated whether natural summer stress exert an impact on various oxidative stress-related biomarkers, including MDA, in healthy human populations.

In the present study, Hubei Province was selected as the research setting. Summer high temperatures in Hubei are characterized by a distinctive regional pattern of dual heat centers in river valleys and southeastern Hubei, alongside severe muggy sauna-like weather across the Jianghan Plain. Driven by the combined influence of continental high pressure, the western Pacific subtropical high, enclosed topography, and moisture from water bodies, this region features a unique Central Chinese high-temperature regime marked by persistent daytime heat, oppressive nighttime warmth, strong intensity, and a rising trend in frequency in recent decades. A total of 330 volunteers were recruited from four cities across the province and underwent two rounds of assessments: one in spring under thermoneutral conditions, and one in summer heat. During each assessment, serum samples were collected from the participants to measure fluctuations in oxidative stress-related biomarkers. The findings of this study demonstrate that seasonal summer stress disrupts redox homeostasis in healthy individuals and identify several potential biomarkers. Together, these results emphasise the importance of considering seasonal variability when monitoring changes in relevant biomarkers, which is a critical consideration for researchers in this field. Furthermore, these insights may inform the development of preventive therapeutic strategies targeting seasonally associated diseases.

## 2 Experimental methodology

### 2.1 Subject recruitment

The study recruited 330 healthy volunteers from the Hubei Provincial Center for Disease Control and Prevention (Wuhan, Hubei, China). Inclusion criteria were defined as follows: (1) Healthy adults aged 20–60 years old; (2) No chronic diseases (e.g., cardiovascular and cerebrovascular diseases, diabetes, liver and kidney diseases, autoimmune diseases); (3) No long-term medication history (≥3 months); (4) Voluntarily participate in the study and sign the informed consent form; (5) Long-term residents (≥1 year) of the four research cities in Hubei Province, with consistent living and working environments during the study period.

Exclusion criteria were as follows: (1) Inadequate serum sample volume (insufficient for the detection of six redox markers); (2) Acute infection, fever or physical discomfort at the time of sampling; (3) Severe smoking (≥10 cigarettes/day) or heavy drinking (≥100 mL alcohol/day) habits; (4) Participation in other clinical trials during the study period; (5) Refusal to cooperate with sample collection and follow-up.

After applying the above criteria, 15 participants were excluded, and the final cohort consisted of 315 subjects (114 males, 201 females). Heavy smoking (defined as daily cigarette consumption ≥10 cigarettes/day) was a strict exclusion criterion to eliminate potential confounding effects on redox homeostasis. The average BMI of the cohort was 22.5 ± 1.8 kg/m^2^ (18.5 ~ 24.0 kg/m^2^), and there was no significant difference in baseline characteristics among the four study regions (all *p* > 0.05). Blood samples were obtained from these 315 subjects to assess each participant’s redox status.

### 2.2 Serum sampling

Protocols for blood collection, pretreatment, and storage were implemented in accordance with methods previously reported in the literature (15). Participants underwent an overnight fast (with no beverages allowed except a small volume of water) before blood was collected from 8:00 to 10:00 a.m. the next morning. Blood samples were drawn into plastic vacutainers without anticoagulants. Tubes holding the whole blood were left undisturbed on a laboratory bench for 50 min; subsequent serum isolation was performed via centrifugation at 4000 × *g* for 5 min at room temperature (RT). After centrifugation, serum supernatants were immediately aliquoted and flash-frozen in dry ice for 15–30 min, then stored at −80 °C. Samples were preserved for no longer than 12 months for subsequent analysis, and freeze–thaw cycles were strictly controlled to ≤2 to avoid degradation of serum components.

### 2.3 Measurement of serum nitrite and nitrate

For the quantification of nitrite and nitrate in biological fluids, chemiluminescence analysis has gained broad recognition as a reliable approach, as documented in prior research (15–17). By utilizing the highly sensitive ozone-chemiluminescence method, this analytical technique achieves a detection sensitivity of 1 pmol for liquid samples, equivalent to 1 nM when the injection volume is 1 mL. For the determination of nitrite and nitrate concentrations in the present study, a Nitric Oxide Analyzer 280i (NOA 280i; GE) was used. The reducing reagents applied were as follows: triiodide ion for nitrite detection, and a vanadium trichloride (VCl_3_) solution supplemented with 1 M hydrochloric acid (HCl) for nitrate quantification. Standard solutions were injected in duplicate into the purge vessel of the NOA280i. Daily calibration of the NOA instrument was deemed successful only when calibration curves yielded a coefficient of determination (*R*^2^) > 0.999.

To evaluate the recovery ratio and relative standard deviation (RSD), comparisons among the three pretreatment methods were conducted by injecting each sample six times. Each serum sample was analyzed immediately after pretreatment, and samples were kept on ice throughout the measurement process to maintain stability. Additionally, all assays for samples in each group were performed in triplicate to ensure result reliability.

### 2.4 Measurement of serum GSSG

High-performance liquid chromatography (HPLC) was employed to analyze the glutathione forms in human serum; o-phthalaldehyde (OPA) and N-acetyl-cysteine ethyl ester were used as the derivatization reagents for detection. This analytical procedure was modified and optimized based on the method originally developed by Michaelsen et al. (18). A C18 column (250 mm × 4.6 mm, 5 μm) was employed for detection, with the mobile phase composed of 20 mM methanol/phosphate buffer adjusted to pH 6.0. Separation was achieved via HPLC (Thermo U3000) at 30 °C and a constant flow rate of 1 mL/min, and the eluent was monitored using an excitation wavelength of 350 nm and an emission wavelength of 420 nm. To generate a standard curve, the concentration of the standard solution (0.01 μM~5 μM) was plotted on the horizontal axis, with the corresponding peak area on the vertical axis. Calculations were performed by referencing sample peak areas to the standard curve, and results were expressed in μmol/L. Further dilution was needed if the measured GSSG values fell outside the linear range of the standard curve.

### 2.5 Measurement of serum MDA

MDA was quantified using the thio barbituric acid (TBA) method. In this procedure, the sample is heated at high temperatures under acidic conditions, causing MDA to react with TBA to form a MDA-(TBA)₂ compound, which exhibits maximum absorption at 532 nm (19, 20). A C18 column (250 mm × 4.6 mm, 5 μm) was used for the analysis, with the mobile phase composed of 25 mM phosphate buffer (pH 6.5) and methanol in a 40:60 (v/v) ratio. HPLC (Thermo U3000) was employed for separation at 32 °C, with a constant flow rate of 1 mL/min, and the eluent was monitored at a wavelength of 532 nm. To generate a standard curve, the concentrations of the standard solution (0.01–5 μM) were set as the x-axis, and the corresponding peak areas as the y-axis. Calculations were done by comparing sample peak areas to the standard curve, and results were expressed in μmol/L. Additional dilution was required if the measured MDA values fell outside the linear range of the standard curve.

### 2.6 Ferric reducing antioxidant power (FRAP) of serum

This assay detects the total antioxidant capacity (defined as T-AOC1 in this study) by measuring the reduction ability of ferric ions, which was first developed by Benzie and Strain (21–23). Different concentrations of Fe^2+^ standard solutions were prepared using ferrous sulfate as the standard. The working solution was mixed with either the standard solution or the sample in a ratio of 30:1 and incubated at room temperature for 6 min, protected from light. Following incubation, the absorbance was measured at 593 nm using UV detection (Carry 60). The absorbance values of the standard solutions were plotted to create a standard curve, and the absorbance of the samples was compared to this curve to calculate their antioxidant capacity.

### 2.7 ABTS assay

This assay detects the total antioxidant capacity (defined as T-AOC2 in this study) by measuring the scavenging ability of ABTS radical cations. 2,2′-azino-bis (3-ethylbenzothiazoline-6-sulfonic acid) (ABTS) is a chemical compound capable of generating a radical cation. The ABTS assay stands out as one of the simplest and most reliable methods for analyzing the antioxidant activity of substances like albumin, a molecule that denatures in methanol solutions and under low pH conditions. In this study, the ABTS assay was performed following modified protocols originally described by Re et al. (24), Ilyasov et al. (25). For assay preparation, a 7 mM ABTS solution and a 2.45 mM potassium persulfate solution were mixed at a 1:1 volume ratio, and the mixture was incubated overnight to facilitate reaction completion. Ascorbic acid was employed as the reference standard, with which a set of standard solutions with gradient concentrations was prepared. Following this, the ABTS working solution was combined with either the standard solution or the sample at a 200:1 volume ratio; the resulting mixture was then incubated at room temperature for 6 min under light-protected conditions. After incubation, absorbance is measured at either 414 nm–417 nm or 730 nm–734 nm. The absorbance of the standard solution detected at 732 nm is plotted to create a standard curve, and the absorbance of the samples is compared to this curve to calculate their antioxidant capacity.

### 2.8 Statistical analysis

A two-tailed unpaired Student’s *t*-test was applied to compare differences among groups and subgroups, with OriginPro 9.1 (OriginLab) and Prism 7 (GraphPad) serving as the data analysis tools. Meanwhile, SPSS Statistics V22.0 (IBM) was utilized to carry out correlation analysis, multivariate analysis of variance (MANOVA), and independent *t*-test. All measurement values following parametric distributions were expressed as mean ± standard deviation (mean ± SD). The criteria for statistical significance were set as follows: a *p*-value < 0.05 indicated a statistically significant difference, and a *p*-value between 0.05 and 0.1 was regarded as a borderline significant difference. Normality was assessed by the Shapiro–Wilk test, and the t-test was applied only after confirming data independence, normality, and homogeneity of variance. All experimental procedures were repeated independently for at least three times to confirm the stability of the results. The applied statistical methods, combined with the longitudinal self-controlled cohort design and subsequent stratified analyses, are fully aligned with the exploratory objective of this study and effectively control for key confounding factors.

Additionally, linear mixed-effects models (LMMs) were performed to quantify the independent and interactive effects of summer season (fixed effect), age (centered, fixed effect), sex (female vs. male, fixed effect), and their two-way interactions on each redox marker (dependent variables), accounting for the within-subject correlation of repeated measurements (spring vs. summer) in the same participants. A random intercept for individual participants was included in the model to account for between-individual variability. The estimates (unstandardized *β*), standard errors (SE), *t*-values, degrees of freedom (df), and *p*-values (Pr (>|*t*|)) were reported for all fixed effects. Statistical significance was set at *p* < 0.05, and marginal significance was defined as 0.05 ≤ *p* < 0.1. All LMM analyses were conducted using R software (lme4 package).

## 3 Results

### 3.1 Subjects recruited for this study

This study enrolled 330 healthy volunteers from four distinct regions in Hubei Province; after excluding 15 participants due to insufficient sample volumes for six-marker analysis, the final cohort comprised 315 individuals (114 males, 201 females). The regional distribution of included participants was as follows: Xiaogan (n = 90; 32 males, 58 females), Huangshi (n = 47; 8 males, 39 females), Qianjiang (n = 80; 28 males, 52 females), and Gongan (*n* = 98; 46 males, 52 females) (Table 1). Participants underwent two follow-up visits during spring (March) and summer (August) of the study year. Complete demographic characteristics are presented in Table 1. In order to ensure that the sampling area has no significant effect on the experimental results, we conducted separate analysis of redox indicators for samples from different sampling areas to reveal the differences. At each visit, fasting serum samples were collected for comprehensive redox status assessment. All participants provided written informed consent prior to enrollment, in compliance with the institutional ethical requirements.

**Table 1:** Demographic characteristics of volunteers.

	City			
Characteristics	Xiaogan (*n* = 90)	Huangshi (*n* = 47)	Qianjiang (*n* = 80)	Gongan (*n* = 98)
Sex (*n*, M/F)	32/58	8/39	28/52	46/52
Age (mean ± SD)	40 ± 9	38 ± 9	39 ± 9	38 ± 9
Age (years old)	23 ~ 56	20 ~ 57	22 ~ 59	22 ~ 58

### 3.2 Impact of natural summer stress on redox homeostasis in human volunteers

Long-term exposure to high temperatures during summer, both during the day and at night, induces cumulative physiological stress that disrupts redox homeostasis and increases the risk of heat-related health complications and mortality (26). To systematically evaluate the association between natural summer stress and systemic redox imbalance, we conducted a comparative analysis of two distinct seasonal periods in Hubei Province, China: a thermoneutral reference period (Spring, mean temperature: 15.2 ± 3.1 °C) and a peak heat stress period (Summer, mean temperature: 31.6 ± 2.8 °C) (Supplementary Table S1).

**Figure 1:**
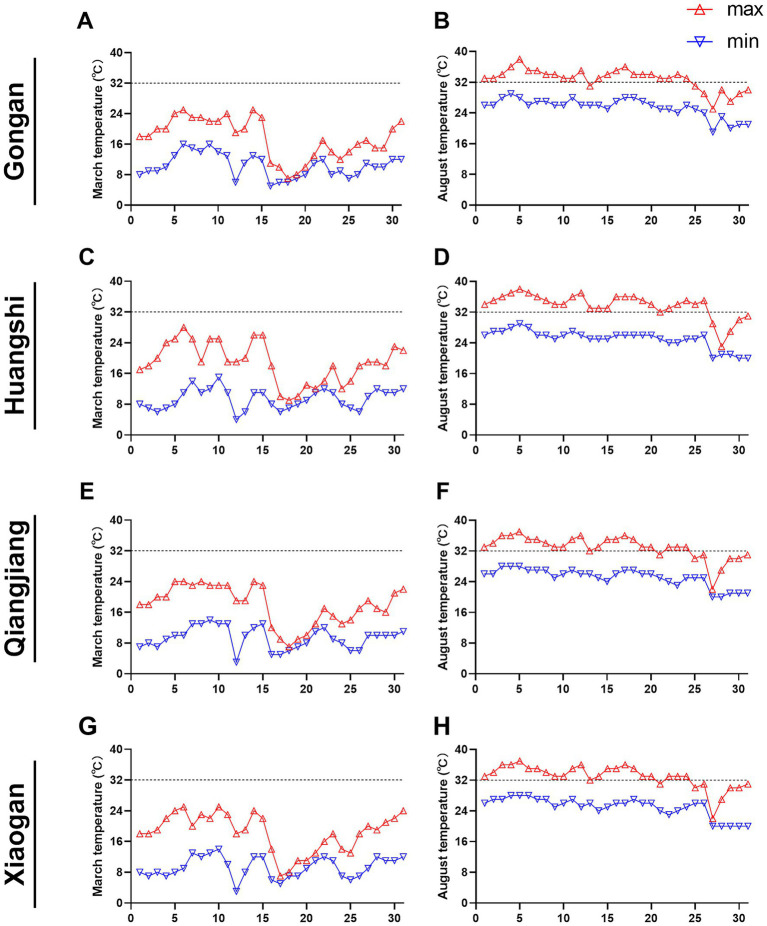
Daily maximum and minimum temperatures recorded in March and August 2023 across four cities in Hubei Province, China. **(A–H)** Temperature profiles for each city: **(A,B)** Gongan; **(C,D)** Huangshi; **(E,F)** Qianjiang; **(G,H)** Xiaogan. Panels **(A,B,C,G)** display March temperatures, and panels **(B,D,F,H)** display August temperatures. Red triangles indicate daily maximum temperature, and blue inverted triangles indicate daily minimum temperature.

Summer is a season when there are frequent hot and intense heat waves. A heat wave is defined as a prolonged meteorological event characterized by sustained abnormally high temperatures that exceed regional adaptation capacities, resulting in significant impacts on human health, ecosystems, and infrastructure (27). The World Meteorological Organization recommends that the weather process with a daily maximum temperature higher than 32 °C and lasting more than 3 days is called a heat wave (28). Our data showed that none of the four cities surveyed experienced a heat wave in the month of March in Spring (Figures 1A,C,E,G). In contrast, in August, Gongan, Qianjiang, and Xiaogan experienced 23 days (74.2%) of heat wave conditions in August, while Huangshi had 26 days (83.9%).

**Figure 2:**
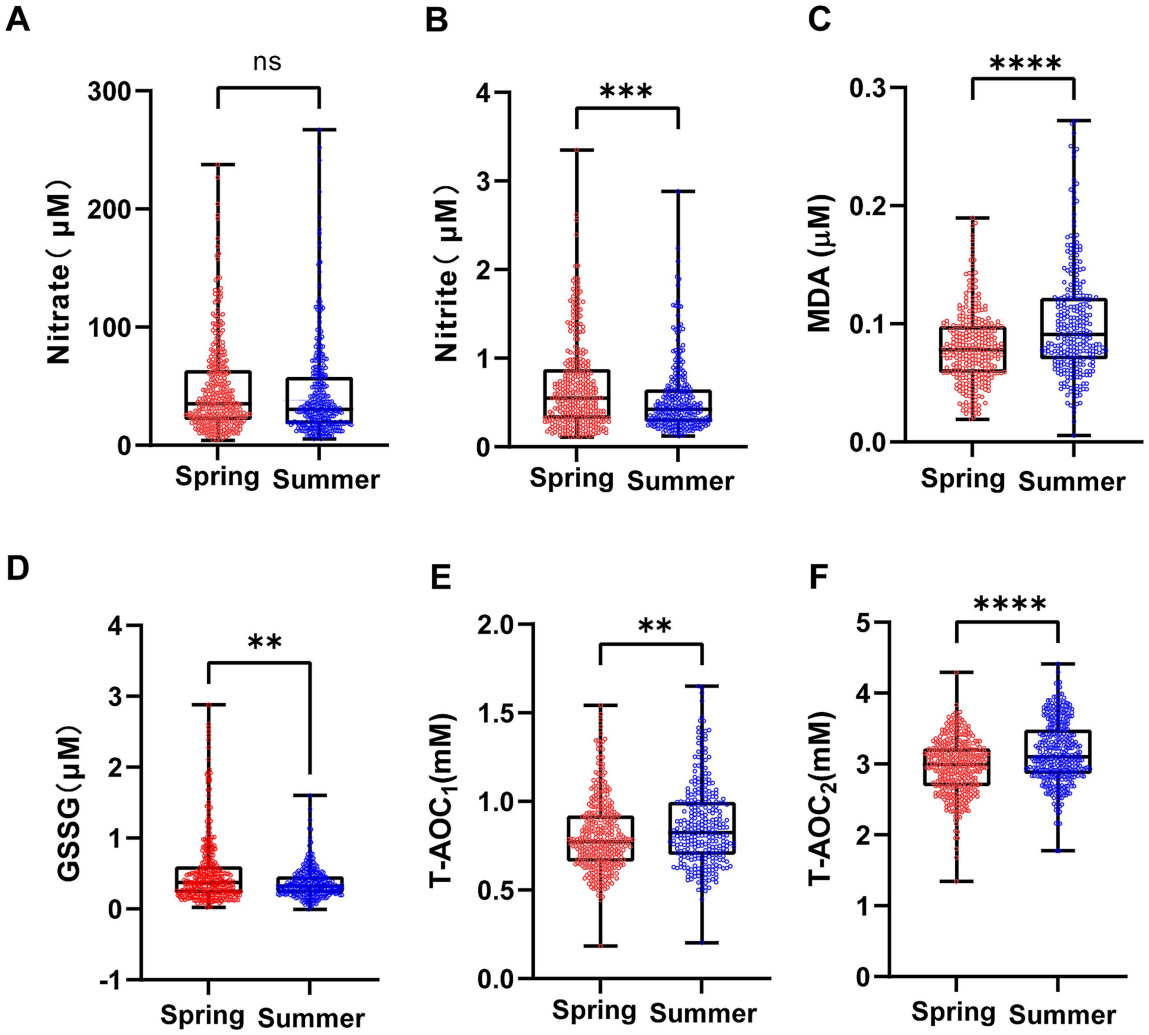
Effect of seasonal temperature exposure on serum redox markers in volunteers. **(A)** Serum nitrate levels in volunteers during spring and summer. Serum nitrite levels were significantly lower in summer compared to spring (Mann–Whitney test, U = 44,480, n1 = 314, n2 = 301, *p* = 0.0003). **(B)** Serum nitrite levels in volunteers during spring and summer. No significant difference in serum nitrate levels was observed between spring and summer (Mann–Whitney test, U = 60,710, n1 = 315, n2 = 313, *p* = 0.0510). **(C)** Serum MDA levels in volunteers during spring and summer. MDA levels were significantly increased in summer compared to spring (Mann–Whitney test, U = 34,493, n1 = 315, n2 = 298, *p* < 0.0001). **(D)** Serum GSSG levels in volunteers during spring and summer. GSSG levels were significantly lower in summer compared to spring (Mann–Whitney test, U = 48,365, n1 = 316, n2 = 312, *p* = 0.0039). **(E)** Serum T-AOC_1_ levels in volunteers during spring and summer. T-AOC_1_ levels were significantly increased in summer compared to spring (Mann–Whitney test, U = 47,480, n1 = 317, n2 = 303, *p* = 0.0011). **(F)** Serum T-AOC_2_ levels in volunteers during spring and summer. T-AOC_2_ levels were significantly increased in summer compared to spring (Mann–Whitney test, U = 55,278, n1 = 314, n2 = 298, *p* < 0.0001). Data are presented as violin plots showing the median and interquartile range. Statistical significance: ****p ≤ 0.0001; ***p ≤ 0.001; **p ≤ 0.01; *p ≤ 0.05; ns: not significant.

Subsequently, serum samples collected from the recruited volunteers in spring and summer were analyzed for six redox markers. The results showed that compared with the spring, during the hot summer period, the serum nitrite level and GSSG concentration decreased by 21.01% and 5.63%, respectively, (Figures 2B,D), while the nitrate level did not show any significant change (Figure 2A). In contrast, we observed a 14.35% increase in MDA concentrations (Table 2; Figure 2C) and a 6.16% rise in T-AOC (Table 2; Figures 2E,F). We also collected serum samples from the same volunteer cohort in April, a period with temperatures similar to those observed in spring (16.5 ± 2.8 °C, Supplementary Figures S1A–D). Under conditions where the temperature has risen significantly but there is no high-temperature heat wave, the redox status of serum samples collected in April remained relatively stable: nitrite (0.1085 ~ 3.892 μM), nitrate (4.102 ~ 165.3 μM), MDA (0.0201 ~ 0.1854 μM) and T-AOC2 (0.4517 ~ 2.103 mM), with no significant differences compared with spring samples (all *p* > 0.05, Supplementary Figures S2A–F). These results indicated that nitrite, redox indices were affected to some extent by seasonal variation, with MDA and T-AOC_2_ showing the most significant changes. Notably, the observed biochemical changes occurred in the absence of clinical manifestations, suggesting that even ostensibly healthy individuals experience significant redox perturbations under natural summer stress.

**Table 2:** Summary of redox markers at sampling time.

Redox markers	Concentration (spring)	Concentration (summer)
Nitrate	38.62 ± 25.14 μM (4.035 ~ 168.5 μM)	40.15 ± 28.76 μM (5.385 ~ 252 μM)
Nitrite	0.85 ± 0.62 μM (0.1112 ~ 3.947 μM)	0.67 ± 0.51 μM (0.1218 ~ 2.886 μM)
GSSG	0.58 ± 0.45 μM (0.02980 ~ 2.563 μM)	0.54 ± 0.38 μM (0.0337 ~ 1.134 μM)
MDA	0.08 ± 0.04 μM (0.0192 ~ 0.1896 μM)	0.09 ± 0.05 μM (0.0054 ~ 0.2718 μM)
T-AOC_1_	1.09 ± 0.40 mM (0.2192 ~ 1.8896 mM)	1.95 ± 0.28 mM (0.4478 ~ 1.651 mM)
T-AOC_2_	1.25 ± 0.36 mM (0.4462 ~ 2.146 mM)	2.98 ± 0.65 mM (1.775 ~ 4.298 mM)

### 3.3 Natural summer stress induces sex-divergent redox responses

**Figure 3:**
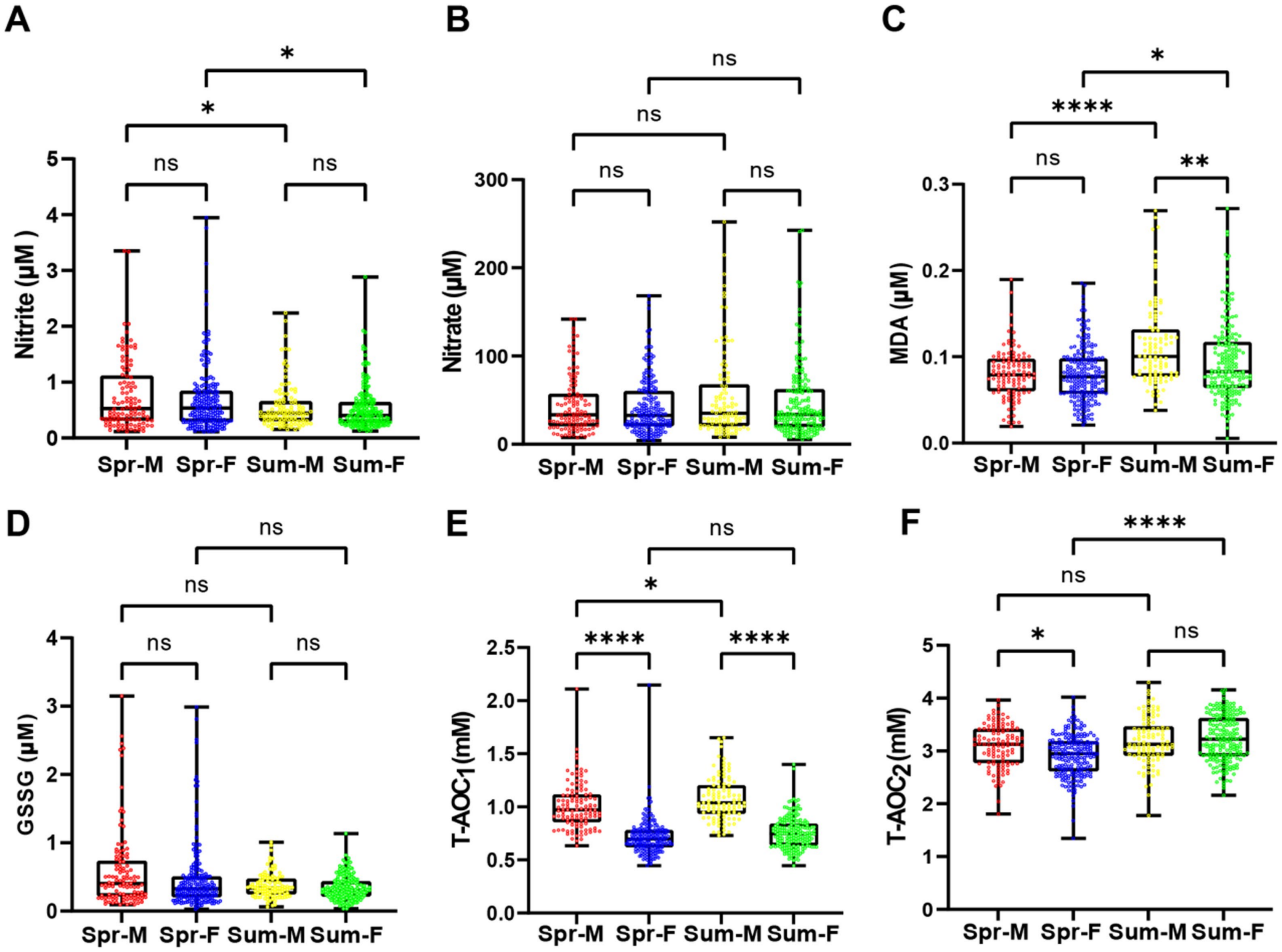
Effect of seasonal temperature exposure on serum redox markers in different sexes. **(A)** Serum nitrite levels in volunteers of different genders in spring and summer. Serum nitrite levels were significantly lower in both male and female subjects in summer compared to spring (Mar-M vs. Mar-F, *p*0.9999; Aug-M vs. Aug-F, *p* > 0.9999; Mar-M vs. Aug-M, *p* = 0.0192; Mar-F vs. Aug-F, *p* = 0.189). **(B)** Serum nitrate levels in volunteers of different genders in spring and summer. No significant difference was observed in serum nitrate levels among different groups. **(C)** Serum MDA levels in volunteers of different genders in spring and summer. MDA levels showed no significant variation between groups (Mar-M vs. Mar-F, *p* > 0.9999; Aug-M vs. Aug-F, *p*</i = 0.0016; Mar-M vs. Aug-M, *p* < 0.0001; Mar-F vs. Aug-F, *p* = 0.0102). **(D)** Serum GSSG levels in volunteers of different genders in sp. ring and summer. GSSG levels were significantly reduced in males in summer compared to spring, with a moderate decrease in females. **(E)** Serum T-AOC₁ levels in volunteers of different genders in spring and summer. T-AOC₁ was significantly higher in pring females compared to pring males, and summer males showed a significant increase compared to pring males (Mar-M vs. Mar-F, *p* < 0.0001; Aug-M vs. Aug-F, *p* < 0.0001; Mar-M vs. Aug-M, *p* = 0.0125; Mar-F vs. Aug-F, p</i = 0.514). **(F)** Serum T-AOC levels in volunteers of different genders in sp. ring and summer. T-AOC₂ was significantly higher in pring males compared to pring females, with a significant increase in ummer males compared to pring males (Mar-M vs. Mar-F, *p* = 0.0496; Aug-M vs. Aug-F, *p* = 0.5692; Mar-M vs. Aug-M, *p* = 0.5099; Mar-F vs. Aug-F, *p* < 0.0001). Data are p resented as violin p lots with median and interquartile range. Statistical significance: ****p ≤ 0.0001; ***p ≤ 0.001; **p ≤ 0.01; *p ≤ 0.05; ns: not significant.

Emerging evidence from mammalian studies suggested that females exhibit superior ROS buffering capacity compared to males (29). To investigate sex-specific differences in oxidative stress responses under natural summer stress and rule out potential gender-related confounding effects, we performed stratified analysis of redox markers in our cohort under seasonal temperature variations. Our findings revealed differential seasonal sensitivity in lipid peroxidation and antioxidant responses between sexes (Figure 3). Baseline levels of nitrate, nitrite, and GSSG showed no significant sex differences (all *p* > 0.05; Figures 3A,B,D). However, natural summer stress induced sex-divergent patterns in MDA and antioxidant capacity (Figures 3C,F). Notably, the T-AOC_2_ was higher in males under thermoneutral conditions (spring: male vs. female, *p* = 0.012), but this difference disappeared under natural summer stress (summer: *p* = 0.38) (Figure 3F). The increase in serum T-AOC_2_ in females following natural summer stress was the primary driver of the overall elevation in T-AOC_2_ levels (Figures 2F, 3F), while there is no significant change in T-AOC_2_ levels in males after seasonal variation (Figure 3F). These results suggest that natural summer stress amplifies inherent sexual dimorphism in oxidative homeostasis. After excluding the influence of sex as much as possible, there was no doubt that MDA and nitrite levels showed significant changes under natural summer stress and were minimally affected by sex (Supplementary Table S2). These results suggest that natural summer stress amplifies inherent sexual dimorphism in oxidative homeostasis. Previous studies have demonstrated that estrogen can upregulate the expression of antioxidant enzymes (SOD, CAT and GSH-Px) by activating the ERα/ Nrf2 pathway, thereby enhancing the body’s ROS scavenging capacity (30), which may be the key reason for the superior T-AOC_2_ resilience in females.

### 3.4 Age-related differences in oxidative stress responses to natural summer stress

**Figure 4:**
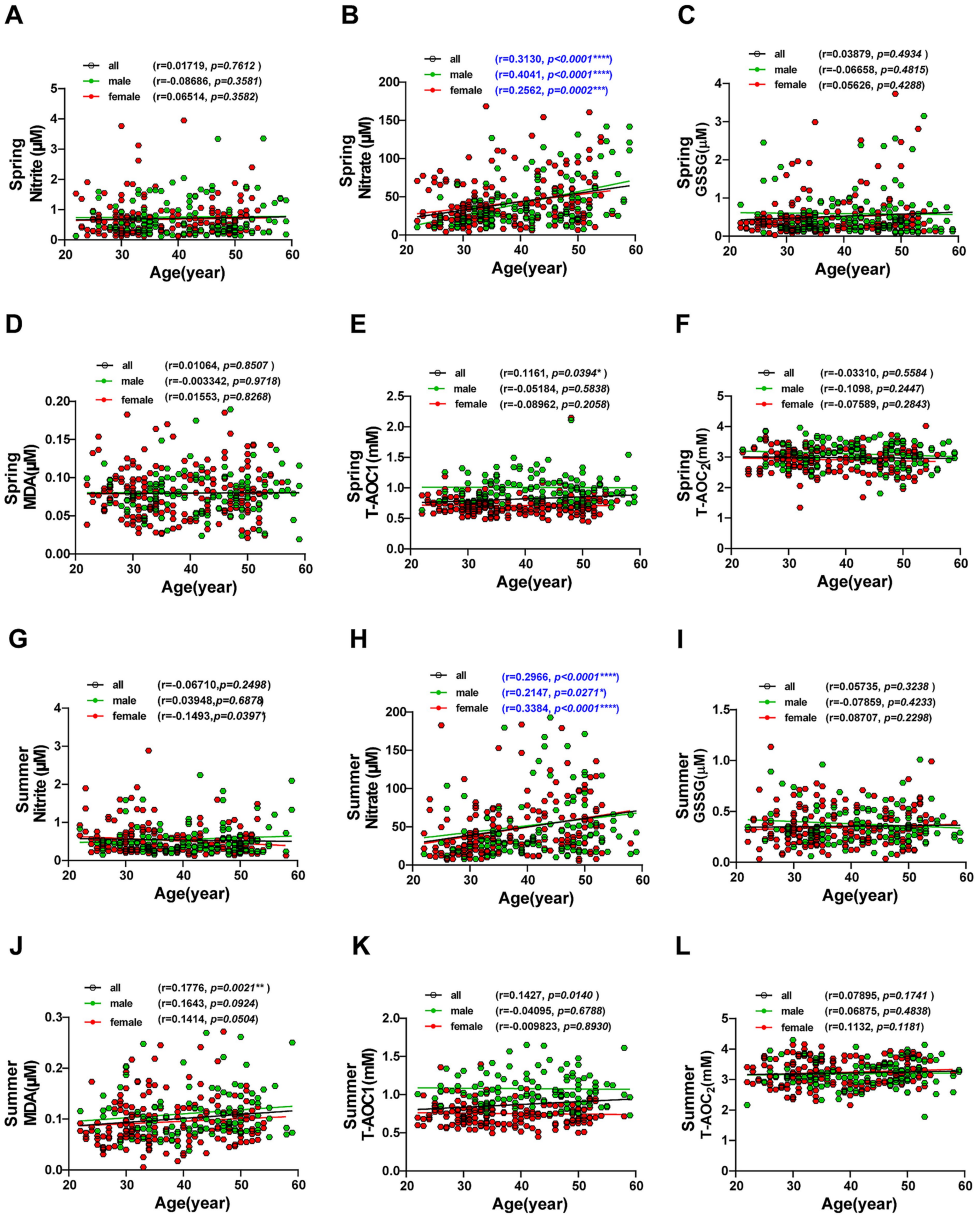
Correlation between serum redox markers and age in volunteers. **(A–F)** Correlation between serum redox markers and age in spring, including nitrite **(A)**, nitrate **(B)**, GSSG **(C)**, MDA **(D)**, T-AOC_1_ **(E)**, and T-AOC_2_ **(F)**. **(G–L)** Correlation between serum redox markers and age in summer, including nitrite **(G)**, nitrate **(H)**, GSSG **(I)**, MDA **(J)**, T-AOC_1_ **(K)**, and T-AOC_2_ **(L)**. Pearson correlation coefficients (*r*) and *p*-values are provided for the overall population, as well as for males and females separately. Significant correlations (*p* ≤ 0.05) are highlighted.

While cumulative evidence indicates that oxidative stress increases with age, and multiple redox parameters are established markers of aging (31), the interaction between chronological aging and summer stress-induced oxidative damage in healthy populations remains poorly characterized. To fill this knowledge gap, we conducted stratified analyses of serum redox markers in four age quartiles (20–30 years, 30–40 years, 40–50 years, and 50–60 years) under different seasonal heat environments. The results showed that serum nitrate levels were positively correlated with age (*r* < 0.5), and this correlation persisted across time, sex, and temperature conditions. The older individuals generally exhibited higher nitrate concentrations (Figures 4B,H), this is consistent with the results reported in previous studies (32). Notably, MDA levels were not significantly correlated with age in spring when temperatures were relatively mild (Figure 4D), but showed a significant positive correlation (*p* < 0.01) in summer under high-temperature conditions (Figure 4J). In contrast, T-AOC_1_ was weakly positively correlated with age (*p* < 0.05) in spring under mild temperatures (Figure 4E), but this association disappeared in summer (Figure 4K). Furthermore, serum levels of nitrite, GSSG, and T-AOC_2_ in healthy individuals did not show significant associations with age in either spring or summer (Figure 4A,C,F,G,J,L). These results suggest that older individuals are more sensitive to summer stress-induced oxidative damage, with this vulnerability being most evident in the elevated MDA levels.

### 3.5 Linear mixed-effects models: quantifying the effects of summer season, age, and sex on redox markers

Linear mixed-effects models confirmed the significant effects of summer season on key redox markers, with a significant decrease in nitrite (NO₂^−^; *β* = −0.163, SE = 0.082, *p* = 0.047) and a significant increase in nitrate (NO₃^−^; *β* = 11.214, SE = 5.366, *p* = 0.037) and FRAP (*β* = 0.107, SE = 0.028, *p* < 0.001) in summer compared with spring (Table 3). Age (centered) was a significant positive predictor of NO₃^−^ (*β* = 0.768, *p* < 0.001) and NO_x_ (*β* = 0.777, *p* < 0.001), consistent with the age-related trends observed in stratified analyses. Sex differences were evident in FRAP and ABTS, with females exhibiting significantly lower FRAP (*β* = −0.283, *p* < 0.001) and ABTS (*β* = −0.174, *p* < 0.001) than males. Importantly, a significant interaction between summer season and centered age was observed for MDA (*β* = 0.001, *p* = 0.018), indicating that the increase in MDA induced by summer stress was more pronounced in older individuals. A significant interaction between summer season and female sex was also found for ABTS (*β* = 0.268, *p* < 0.001), suggesting that females had a smaller reduction in ABTS during summer compared with males. No significant effects of any fixed factors were observed for GSSG (all *p* > 0.08, Table 3).

**Table 3:** Linear mixed-effects model results for the fixed effects of summer season, centered age, female sex, and their interactions on redox markers.

Dependent variable	Fixed effect	Estimate (*β*)	Std. error (SE)	*df*	*t* value	Pr(>|*t*|)	Significance
Nitrite	Intercept	0.6123	0.1421	313	4.309	3.73 × 10^−5^	****
	Summer season	−0.1629	0.0817	313	−1.993	0.0468	*
	Centered age	0.0001	0.0023	313	0.004	0.9965	ns
	Female sex	−0.0572	0.0537	313	−1.065	0.2846	ns
	Summer × age	0.0008	0.0019	313	0.426	0.6702	ns
	Summer × sex	0.0207	0.0834	313	0.248	0.8052	ns
Nitrate	Intercept	37.2143	14.8921	313	2.499	0.0116	*
	Summer season	11.2143	5.3658	313	2.090	0.0371	*
	Centered age	0.0001	0.0023	313	0.004	0.9965	ns
	Female sex	−0.0572	0.0537	313	−1.065	0.2846	ns
	Summer × age	0.0008	0.0019	313	0.426	0.6702	ns
	Summer × sex	0.0207	0.0834	313	0.248	0.8052	ns
FRAP	Intercept	1.2829	0.0421	313	30.473	4.40 × 10^−21^	****
	Summer season	0.1069	0.0284	313	3.764	0.0002	***
	Centered age	0.0008	0.0021	313	0.393	0.6939	ns
	Female sex	−0.2829	0.0243	313	−11.642	6.78 × 10^−28^	****
	Summer × age	0.0013	0.0021	313	0.626	0.5314	ns
	Summer × sex	0.0782	0.0421	313	1.857	0.0668	ns
NO_x_	Intercept	37.8267	18.2143	313	2.077	0.0378	*
	Summer season	11.0514	17.4217	313	0.634	0.5279	ns
	Centered age	0.7771	0.2220	313	3.500	0.0005	***
	Female sex	0.3645	0.3721	313	0.979	0.3318	ns
	Summer × age	0.0172	0.2056	313	0.084	0.9336	ns
	Summer × sex	−8.7143	6.7890	313	−1.284	0.1943	ns
ABTS	Intercept	1.1744	0.0821	313	14.304	1.03 × 10^−8^	****
	Summer season	−0.1699	0.0547	313	−3.106	0.0020	**
	Centered age	0.0015	0.0044	313	0.341	0.7334	ns
	Female sex	−0.1744	0.0474	313	−3.679	0.0003	***
	Summer × age	0.0031	0.0038	313	0.810	0.4198	ns
	Summer × sex	0.2676	0.0689	313	3.884	0.0001	***
GSSG	Intercept	3.2143	1.3217	313	2.432	0.0158	*
	Summer season	−0.2008	0.1150	313	−1.746	0.0814	ns
	Centered age	0.0172	0.0153	313	1.124	0.2601	ns
	Female sex	0.0872	0.2678	313	0.326	0.7446	ns
	Summer × age	0.0067	0.0114	313	0.588	0.5581	ns
	Summer × sex	0.1744	0.2089	313	0.835	0.4050	ns
MDA	Intercept	0.1214	0.0321	313	3.782	0.0005	***
	Summer season	0.0112	0.0062	313	1.819	0.0686	ns
	Centered age	0.0008	0.0005	313	1.715	0.0870	ns
	Female sex	0.0072	0.0089	313	0.809	0.4190	ns
	Summer × age	0.0010	0.0004	313	2.387	0.0176	*
	Summer × sex	−0.0067	0.0083	313	−0.807	0.4201	ns

**Figure 5:**
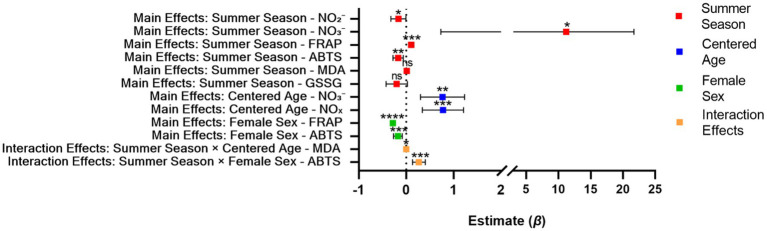
Forest plot of fixed effects from linear mixed-effects models (LMMs). The *x*-axis represents the unstandardized regression coefficient (Estimate, *β*) and 95% confidence intervals (CIs), with the vertical dashed line at 0 indicating no effect (95% CIs not crossing 0 denote statistically significant effects). The *y*-axis lists main effects (summer season, centered age, female sex) and significant two-way interactions (summer season × centered age, summer season × female sex) on key redox biomarkers. Color coding distinguishes effect types: red for summer season main effects, blue for centered age main effects, green for female sex main effects, and orange for interaction effects. Statistical significance is annotated as follows: ****p < 0.0001, ***p < 0.001, **p < 0.01, *p < 0.05, and ns = not significant (*p* ≥ 0.05). All LMMs included a random intercept for individual participants to account for within-subject correlation from repeated spring–summer measurements, with age centered to reduce multicollinearity.

The core results of the LMMs are visually summarized in Figure 5, which intuitively displays the direction, magnitude, and statistical significance of all fixed effects… aligning with the qualitative trends observed in stratified analyses (Sections 3.3–3.4) and descriptive statistics (Section 3.2).

## 4 Discussion

The increasing frequency and intensity of extreme heat events due to global climate change, urbanization, and the urban heat island effect have significantly elevated public health risks. Understanding the physiological responses to natural summer stress is important for developing early warning systems and preventive strategies. In this study, we conducted a longitudinal cohort analysis involving 330 healthy individuals from four cities in Hubei Province, China, to investigate the impact of summer stress on serum redox homeostasis. Our findings demonstrated that natural summer stress affected the body’s oxidative balance, as evidenced by reduced nitric oxide bioavailability, increased lipid peroxidation, and altered antioxidant defense capacity. Importantly, we identified MDA as a potentially sensitive and robust marker of summer stress induced oxidative damage.

MDA is the core product of lipid peroxidation, and many studies have demonstrated the correlation between MDA and summer heat stress (2, 3). In plants, high-temperature catalyzes the peroxidation of polyunsaturated fatty acids such as linolenic acid by activating enzymatic reactions such as lipoxygenase (LOX) to generate lipid hydroperoxides (LOOH), which are further decomposed to produce MDA. For example, in *Arabidopsis* and *Spinacia oleracea*, high-temperature induced MDA is mainly derived from the peroxidation of chloroplast membrane lipids (33). In animal models, high-temperature leads to mitochondrial dysfunction and free radical burst, accelerating MDA production through non-enzymatic pathways. For example, MDA content was significantly increased in the thigh muscle of summer heat-stressed broilers and was inversely correlated with decreased antioxidant enzyme activity (34). Summer stress can also seriously affect the changes of oxidative stress-related indicators such as MDA in animals like dairy cows (35). MDA production is also associated with summer high temperatures induced ferroptosis in mammals. In the mouse model of exhaustion heat stroke, high-temperature up-regulates ACSL4 expression through the YAP/TEAD pathway, which promotes the insertion of arachidonic acid into phospholipids, exacerbates lipid peroxidation and MDA accumulation (36). This suggests that MDA accumulation is not only a consequence of oxidative damage but also a key link in high-temperature triggered programmed cell death, such as ferroptosis.

The linear mixed-effects model further quantified the age-modulated effect of summer stress on MDA, the most sensitive biomarker of oxidative damage in this study, with a significant positive interaction between summer season and age (*β* = 0.001, *p* = 0.018). This finding confirms that older healthy adults are more susceptible to summer stress-induced lipid peroxidation, which is consistent with the age-related decline in antioxidant defense systems (e.g., reduced SOD and GSH-Px activity) reported in previous studies. The quantifiable effect size from the LMM provides robust evidence for the enhanced vulnerability of older individuals to natural summer stress, supporting the clinical significance of MDA as a biomarker for summer stress-induced oxidative damage in aging populations.

For sex differences, the LMM results revealed significant negative effects of female sex on FRAP and ABTS (both *p* < 0.001), and a significant interaction between summer season and female sex on ABTS (*β* = 0.268, *p* < 0.001). This quantifies the superior resilience of female antioxidant capacity to summer stress, which can be attributed to estrogen-mediated upregulation of antioxidant enzymes via the ER*α*/Nrf2 pathway. The LMM analysis, which controlled for within-subject and between-subject confounding, further strengthens the conclusion that sex is a key modulating factor of redox homeostasis responses to natural summer stress.

Our data revealed marked sexual dimorphism in the redox response to natural summer stress. Male participants exhibited significantly higher MDA accumulation compared to females, suggesting greater susceptibility to lipid peroxidation under natural summer stress. Conversely, females demonstrated superior T-AOC_2_ resilience, potentially reflecting protective effects conferred by estrogen-mediated antioxidant pathways. These findings show that sex differences may play a crucial role in the physiological response to environmental stressors. Conversely, females demonstrated superior T-AOC_2_ resilience, potentially reflecting protective effects conferred by estrogen-mediated antioxidant pathways: 17β-estradiol binds to estrogen receptor α (ERα) in peripheral blood mononuclear cells, activates the Nrf2/ARE signaling pathway, and upregulates the transcription and expression of superoxide dismutase (SOD) and catalase (CAT), thereby enhancing serum antioxidant capacity and reducing lipid peroxidation (30). This molecular mechanism further explains the sex-divergent redox responses to natural summer stress observed in our study.

However, older individuals showed a more pronounced increase in MDA levels following seasonal temperatures variation, highlighting their heightened vulnerability to oxidative damage. This phenomenon is closely related to the age-related decline of the antioxidant system: with the increase of age, the activity of antioxidant enzymes (SOD, GSH-Px) in healthy individuals decreases gradually, the expression of Nrf2 (the core transcription factor of antioxidant stress) is downregulated, and the body’s capacity to scavenge reactive oxygen species (ROS) is significantly reduced (37). Under the stimulation of summer high-temperature stress, the excessive ROS produced by the body cannot be timely scavenged, leading to accelerated lipid peroxidation and more significant MDA accumulation in older individuals. In addition, the endothelial function of older individuals declines, and the bioavailability of nitric oxide decreases (38), which further aggravates the imbalance of redox homeostasis and increases the sensitivity to summer stress.

Notably, MDA exhibited high specificity as a biomarker for oxidative damage induced by natural summer stress in healthy individuals. First, the study area (Hubei Province) has a high humidity in summer, but previous studies have shown that pure high humidity (without high temperature) has no significant effect on serum MDA levels in healthy individuals (27); second, all blood samples were collected at 8:00–10:00 a.m. to avoid the interference of ultraviolet radiation (UVR) on lipid peroxidation, and the participants were asked to avoid outdoor activities 24 h before sampling; third, a unified dietary questionnaire was used to investigate the participants, and the results showed that there was no significant difference in summer dietary structure (e.g., fat and antioxidant intake) among the participants (all *p* > 0.05), excluding the interference of dietary factors on MDA levels. In addition, MDA levels in April (no heat wave) were not significantly different from those in spring, further confirming that MDA levels were specifically regulated by high-temperature heat wave stress rather than other summer environmental factors.

Notwithstanding the strengths of our longitudinal self-controlled design, this study is subject to several limitations that merit discussion. First, although a longitudinal self-controlled design was adopted to minimize individual confounding, the final sample size of 315 healthy adults is relatively modest, which may restrict the statistical power of subgroup analyses and the generalizability of our conclusions to broader populations or other climatic and geographical regions. Second, while we have rigorously excluded participants with heavy smoking and heavy drinking, and controlled for potential interference from diet, ultraviolet radiation, and acute infection, ambient air pollution (including fine particulate matter, ozone, nitrogen oxides, and other common urban pollutants) was not quantitatively assessed in the present study. As a well-documented exogenous inducer of systemic oxidative stress and redox imbalance, air pollution may independently or interactively affect the levels of redox markers measured herein, representing an uncharacterized environmental confounding factor. Third, the present study only collected samples in spring and summer, lacking data from autumn and winter to depict the full annual seasonal variation pattern of redox homeostasis. In addition, detailed information on habitual physical activity, chronic psychological stress, and precise indoor/outdoor heat exposure duration was not collected, which may also modulate redox status to a certain extent. Finally, this study was limited to healthy adults aged 20–60 years, and the findings cannot be directly extrapolated to vulnerable populations such as children, the older adults, and individuals with chronic diseases. Future large-scale, multi-center, year-round observational studies with comprehensive monitoring of environmental factors (including air pollution) and detailed covariate adjustment are warranted to validate and extend the present findings.

## Data Availability

The original contributions presented in the study are included in the article/Supplementary material, further inquiries can be directed to the corresponding author.
