# Supplementary material for: Seasonal summer stress affects systemic redox homeostasis: a longitudinal marker analysis in healthy adults
Source: Front Public Health. 2026 May 22;14:1778888. doi: 10.3389/fpubh.2026.1778888 (PMC13236854; doi:10.3389/fpubh.2026.1778888)
Supplement: Supplementary file 1 [file Data_Sheet_1.docx]

**Supplementary Materials**


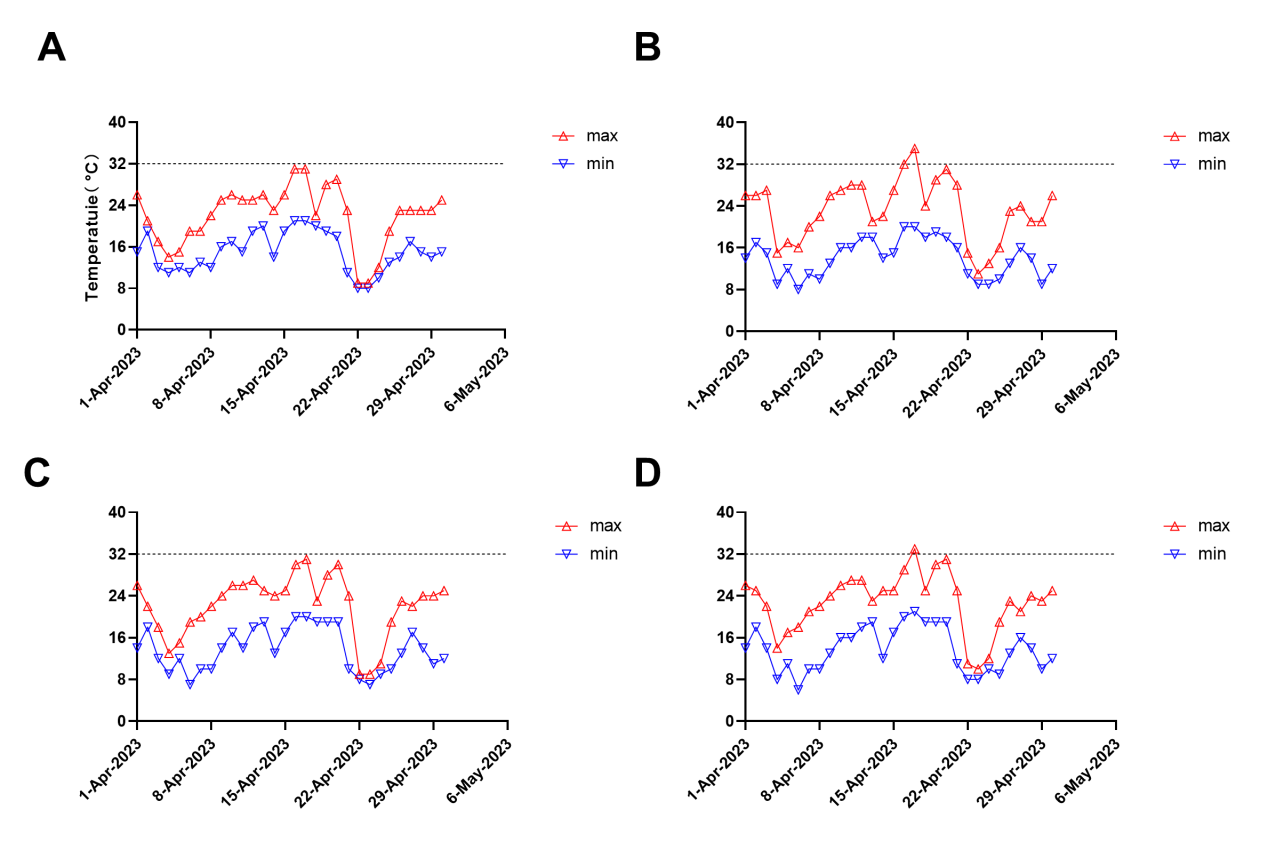


**Figure S1. Recorded temperature every day in April of 2023. A-D**, The temperature distribution in April 2023 for the cities of Gongan (A), Huangshi (B), Qianjiang (C), and Xiaogan (D). Dates with daily maximum temperatures exceeding 32 ° C will be reflected above the dotted line.


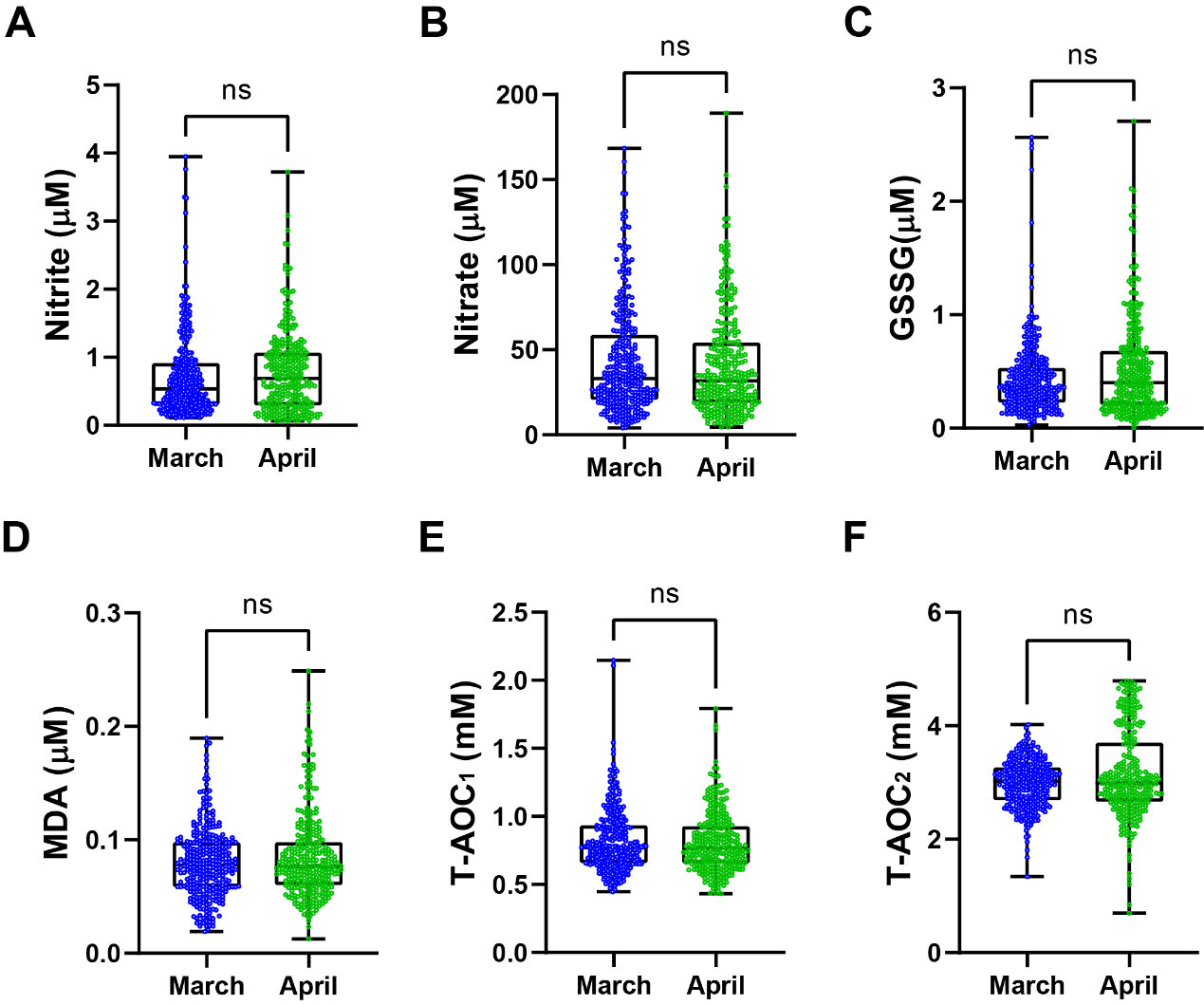
**Figure S2. Effect of temperature in April on serum redox markers in volunteers. (A-F**) there were NO significant changes in serum levels of nitrite (A), nitrate (B), GSSG (C), MDA (D), T-AOC₁ (E) and T-AOC₂ (F) between April and March. Data are presented as violin plots, including median and interquartile ranges. Statistical significance: *****p*≤0.0001; ****p*≤0.001; ***p*≤0.01; *p≤0.05; ns: not significant.

| **City** | **Average Temperature (℃)** | | **Average High Temperature (℃)** | | **Average Low Temperature (℃)** | | **Average Relative Humidity (%)** | |
| --- | --- | --- | --- | --- | --- | --- | --- | --- |
|  | **Mar** | **Aug** | **Mar** | **Aug** | **Mar** | **Aug** | **Mar** | **Aug** |
| Gong'an | 14 | 29.1 | 17 | 31 | 10 | 25 | 65.2 | 78.5 |
| Huangshi | 14 | 29.4 | 18 | 33 | 9 | 25 | 64.8 | 79.2 |
| Qianjiang | 13.6 | 29 | 17 | 32 | 9 | 24 | 66.1 | 77.8 |
| Xiaogan | 13.8 | 29.5 | 18 | 32 | 9 | 24 | 65.5 | 78.1 |

**Table S1.** **Meteorological characteristics of the study region during 2023Table S2. Compared to March, the overall trend of redox changes in August.**

| volunteers | nitrite | nitrate | GSSG | MDA | T-AOC_1_ | T-AOC_2_ |
| --- | --- | --- | --- | --- | --- | --- |
| all | ***↓ | ns | *↓ | ****↑ | **↑ | ****↑ |
| male | *↓ | ns | ns | ****↑ | *↑ | ns |
| female | *↓ | ns | ns | *↑ | ns | ****↑ |

*****p* ≤ 0.0001; ****p* ≤ 0.001; ***p* ≤ 0.01; **p* ≤ 0.05; ns: not significant.
